# Loop-mediated isothermal amplification (LAMP) assay coupled with gold nanoparticles for colorimetric detection of *Trichoderma* spp. in *Agaricus bisporus* cultivation substrates

**DOI:** 10.1038/s41598-024-65971-9

**Published:** 2024-07-05

**Authors:** Mila Djisalov, Ljiljana Janjušević, Vincent Léguillier, Ljiljana Šašić Zorić, Carole Farre, Jamila Anba-Mondoloni, Jasmina Vidic, Ivana Gadjanski

**Affiliations:** 1 BioSense Institute, University of Novi Sad, Novi Sad, Serbia; 2 Micalis Institute, INRAE, AgroParisTech, UMR 1319, Université Paris-Saclay, Jouy en Josas, France; 3 CNRS, Institute of Analytical Science, Université Claude-Bernard Lyon 1, 69100 Villeurbanne, France

**Keywords:** DNA, Biochemistry, Chemistry, Engineering

## Abstract

One of the significant challenges in organic cultivation of edible mushrooms is the control of invasive *Trichoderma* species that can hinder the mushroom production and lead to economic losses. Here, we present a novel loop-mediated isothermal amplification (LAMP) assay coupled with gold nanoparticles (AuNPs) for rapid colorimetric detection of *Trichoderma* spp. The specificity of LAMP primers designed on the *tef1* gene was validated in silico and through gel-electrophoresis on *Trichoderma harzianum* and non-target soil-borne fungal and bacterial strains. LAMP amplification of genomic DNA templates was performed at 65 °C for only 30 min. The results were rapidly visualized in a microplate format within less than 5 min. The assay is based on salt-induced aggregation of AuNPs that is being prevented by the amplicons produced in case of positive LAMP reaction. As the solution color changes from red to violet upon nanoparticle aggregation can be observed with the naked eye, the developed LAMP-AuNPs assay can be easily operated to provide a simple initial screening for the rapid detection of *Trichoderma* in button mushroom cultivation substrate.

## Introduction

Cultivated edible mushrooms are a substantial nutritional source that holds economic significance in various regions across the world^1,2^. Asian countries are the largest mushroom producers contributing up to 76% of the global production (China alone produces about 35%), followed by Europe (17.2%) and United States (5.9%)^3^. The global market value of fresh mushrooms was estimated to US$ 38 billion in 2018^4^, and is expected to increase in future due to the changes in population lifestyle and growing consumers’ food awareness. Mushrooms embody a rich source of healthy nutrients and are a staple in the human diet. They are characterized by a low-calorie content, are devoid of saturated fat and cholesterol, and encompass all essential amino acids. Apart from their nutritional value, the appeal of mushrooms lies in antioxidant activity and therapeutic properties. Additionally, their distinctive taste and unique texture make them an attractive choice for inclusion as a food ingredient or as a substitute for food additives^5–7^, both in traditional food products and in the rising field of alternative proteins.

**Figure 1:**
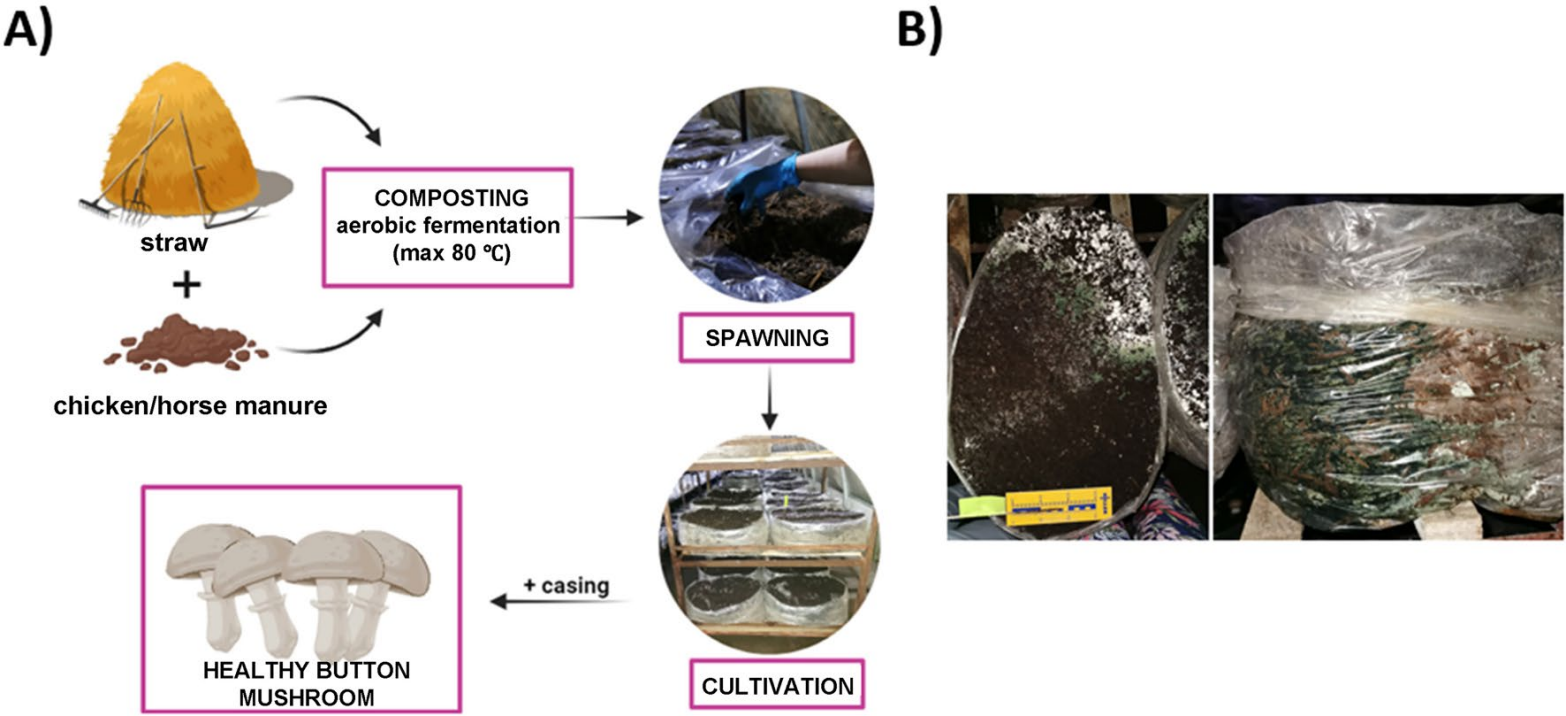
(**A**) Illustration of principle stages in the mushroom cultivation process. (**B**) Images of button mushroom-growing substrate contaminated with *Trichoderma harzianum.*

Modern mushroom farming elicits a number of environmental impacts which can be moderated through the circular economy principles based on upcycling of organic waste in the cultivation process. Button mushrooms (*Agaricus bisporus*) as one of the most consumed species are cultivated using compost, an organic substrate created through a thermophilic microbial process involving crop residues and underutilized wood (or other organic waste), nitrogen-containing additives (typically poultry or horse manure), seed meal, or synthetic nitrogen sources like urea or ammonium nitrate, along with gypsum. During the fruit-bearing phase of button mushroom cultivation, it is essential to add a layer of peat, known as "casing soil," on the surface of the composted substrate. This practice enhances the conditions for optimal mushroom growth as illustrated in Fig. 1A.

During the production of button mushrooms, the emergence of green mold in the nursery stands out as a prevalent issue. This disease, attributed to the rapid invasion of fungi of the genus *Trichoderma* in cultivation bags significantly hinders the fructification of mushrooms (Fig. 1B). When compost or casing are infected with an aggressive species, such as *Trichoderma harzianum,* no mushroom is produced on the infected area since *Trichoderma* feeds on an assortment of other fungi^8^ and utilizes the majority of available nutrients due to its high capability for nutrient uptake^9^. *Trichoderma* spores get introduced into mushroom-growing facilities through contaminated spawn, compost, casing soil and wood. The spread of green mold is facilitated by contaminated tools, substrate, and clothing of employees. Additionally, it can be transmitted through contaminated air and insect vectors, such as sciarid mushroom flies^10^. The early detection of *Trichoderma* spp. on the substrate for growing edible mushrooms including *A. bisporus* as well as other species susceptible to *Trichoderma* infection such as oyster mushroom (*Pleurotus ostreatus*) and shiitake (*Lentinula edodes*), is crucial^11,12^. Early detection is a key step because *Trichoderma* spp. becomes visible only after the formation of dark green spores (Fig. 1B), when it is already too late to prevent the edible mushroom yield loss. *Trichoderma* has been estimated to affect 10–20% of the total worldwide mushroom production every year, resulting not only in economic losses amounting to several billion dollars, but also in diminished food supplies^13–15^.

The main measures to minimize *Trichoderma* contamination in the mushroom farms rely on strict hygiene practices, implementing treatments with disinfectants, and applying fungicides. However, usage of fungicides is not possible in the case of organic production^16–18^. Therefore, *Trichoderma* infestation is particularly detrimental for organic mushroom farms, as they have limited options to counter it. One promising option may be new methods for early *Trichoderma* detection which would allow organic producers to exclude infected substrate from further use, before significant losses in mushroom yield occur. Recently we have reviewed methods for monitoring populations of *Trichoderma* in mushroom-farming conditions^10^. Briefly, traditional methods for detection and identification of *Trichoderma* species in soil are based on isolation techniques using selective media. Unfortunately, this approach is laborious and not adapted for the fastidious nature of some strains. Immunological tests are not adapted for soil analysis because isozyme analysis and serology are nonspecific at the isolate level for *Trichoderma* species. Finally, only a few studies have been published about PCR-based methods to target *Trichoderma* species^19–21^. Although PCR-based assays are sensitive and nucleic acid genetic markers can be highly specific, this approach may provide false-negative responses because of the sensitivity of DNA polymerase to the inhibitors present in soil and to low quantity of *Trichoderma* genomic DNA when isolated in the early phase of disease.

Given the extent of yield loss in mushroom production caused by the onset of green mold disease, there is an urgent need for a new method that will enable early, rapid, and specific detection of *Trichoderma* spp., especially during organic cultivation of button mushrooms. Here, we report a novel loop-mediated isothermal amplification (LAMP) assay coupled with gold nanoparticles (AuNPs) for highly sensitive detection of green mold in *A. bisporus* cultivation substrates (casing soil and compost). LAMP is an isothermal amplification technique that shows a higher degree of specificity and sensitivity compared to PCR due to the larger number of target-specific primers and exceptional resistance to contaminants that inhibit PCR reaction^22,23^. AuNPs were used to allow a colorimetric read-out of the LAMP reaction indicating the presence or absence of *Trichoderma* species. Due to its specificity and simplicity to perform, we expect the developed method of detection of green mold disease in its early phases to be of high value for all mushroom producers.

## Results and discussion

### LAMP assay in naturally infected samples

Before conducting LAMP reactions, primer set specificity for the chosen target *tef1* gene was tested in silico for potential cross-reaction with other species (Supplementary Data S1). The *tef1* gene was chosen as a secondary fungal DNA barcode for *Trichoderma* species. Additionally, to confirm that the developed LAMP primers are genus-specific, primer specificity was tested in silico by using *tef1* genes originated from different *Trichoderma* species (Supplementary Data S2). The position and direction of all LAMP primers within the *tef1* gene are shown in Supplementary Fig. S1. The multiple sequence alignment in Supplementary Data S1 shows that there was no similarity with fungal and bacterial species widely represented in the soil materials such as compost and casing soil.

**Figure 2:**
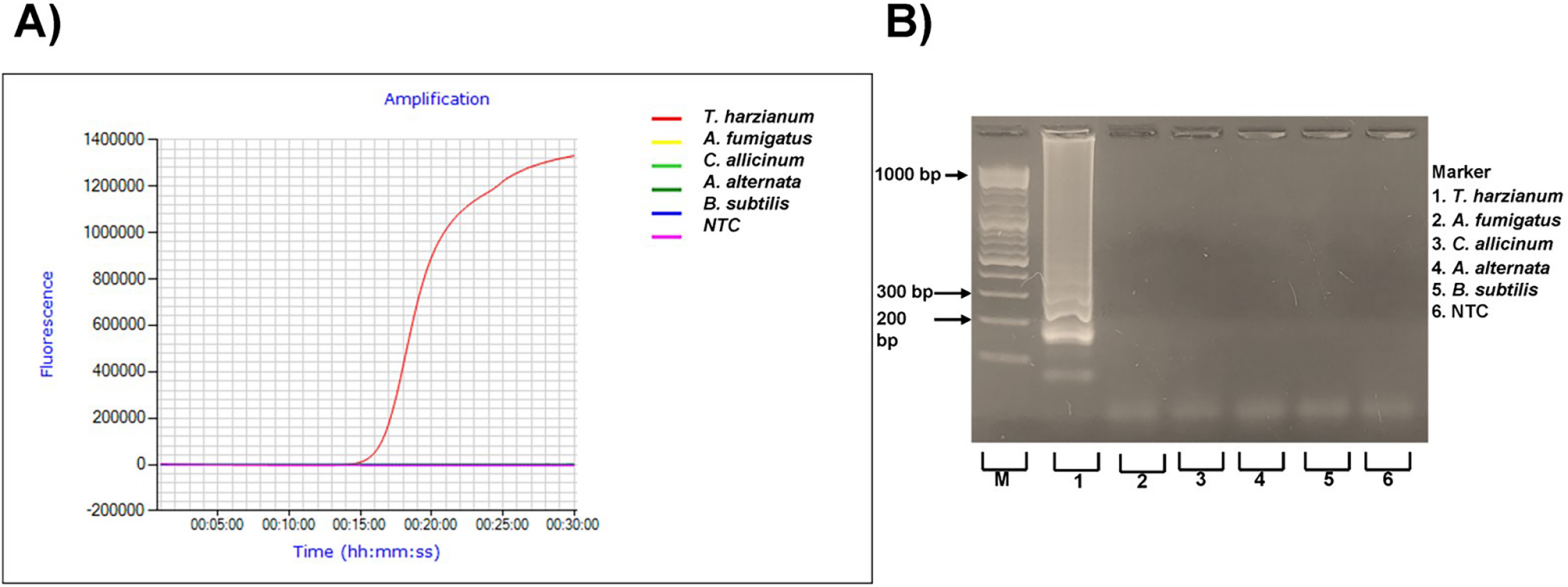
Validating the specificity of LAMP reaction for *Trichoderma harzianum* detection: (**A**) by using Genie® III instrument and (**B**) on 2% agarose gel. Concentrations of gDNAs extracted from *Trichoderma harzianum*, *Aspergillus fumigatus*, *Alternaria alternata, Cladosporium allicinum* and *Bacillus subtilis* were: 75 ng/µL, 72.30 ng/µL, 28.67 ng/µL, 18.66 ng/µL, 26.01 ng/µL, respectively.

The specificity of the LAMP primer set was then experimentally validated using gDNAs extracted from *T. harzianum* (as a model for highly aggressive *Trichoderma* spp*.*) isolated from the substrate samples, non-target fungal strains, and soil-borne bacterium *Bacillus subtilis*. The conditions for LAMP detection of *tef1* were optimized to complete in 30 min at 65 °C (Fig. 2A). The amplification was confirmed through positive bands in the 2% gel electrophoresis (Fig. 2B, Supplementary Fig. S8). No band was observed on negative controls confirming that positive LAMP reaction occurred exclusively when *Trichoderma* gDNA was used as the template. These LAMP conditions were applied for development of the LAMP-AuNPs colorimetric assay for *Trichoderma* detection.

One of the most critical parameters in detection assays is the sensitivity. To check the sensitivity of the LAMP method, LAMP primers were used to amplify the target *T. harzianum* gDNA from tenfold serial dilutions (Supplementary Fig. S7).

### AuNPs production and characterization

**Figure 3:**
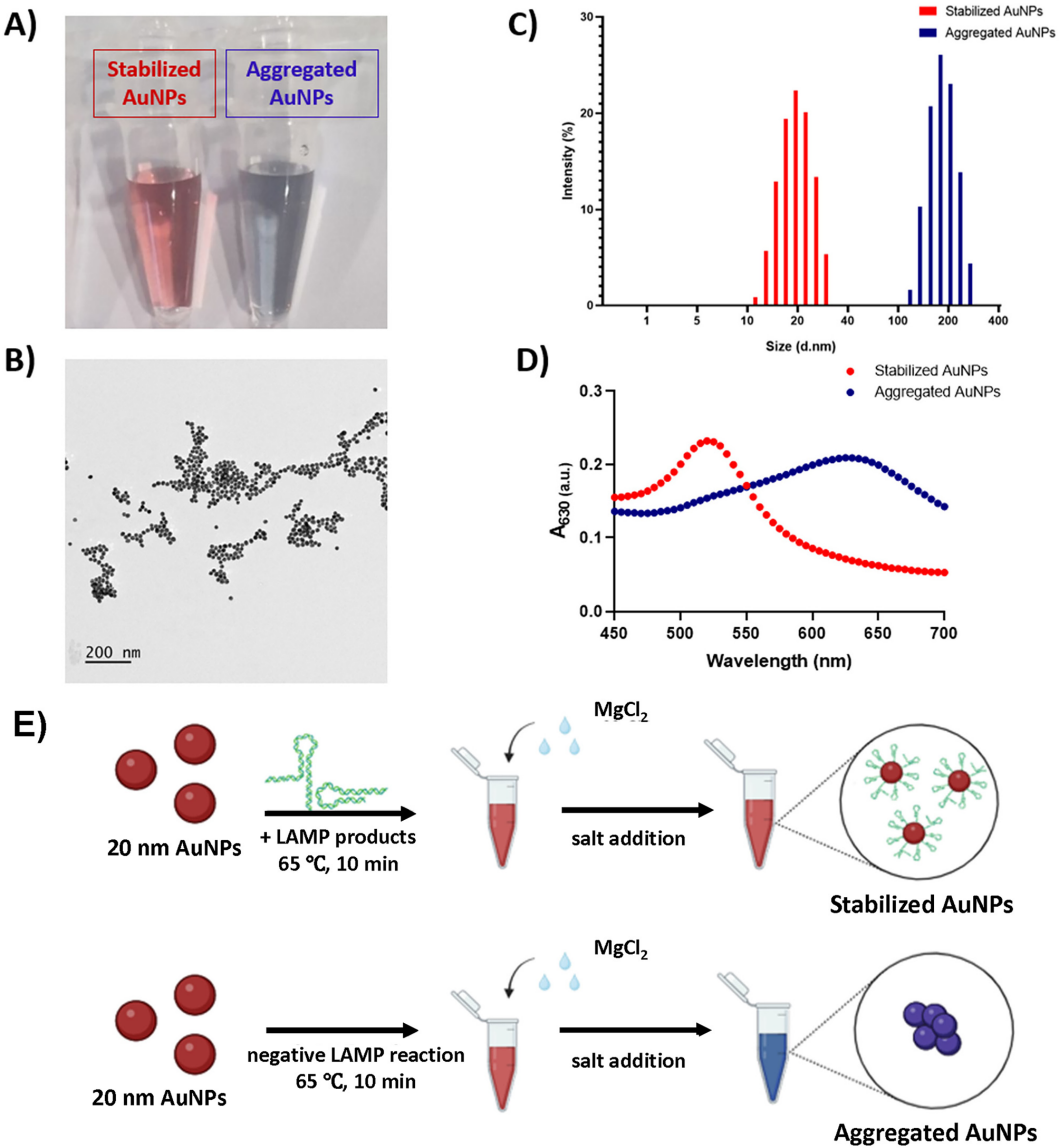
Salt control aggregation of AuNPs. (**A**) Color differences between stable (no MgCl_2_ added) and aggregated AuNPs (5 mM MgCl_2_ added); (**B**) Transmission electron microscopy (TEM) of synthetized AuNPs; (**C**) Dynamic light scattering (DLS) of stabilized and aggregated AuNPs and (**D**) UV–Vis spectra of stabilized and aggregated AuNPs; (**E**) Schematic presentation of the colorimetric sensor coupled with loop-mediated isothermal nucleic acid amplification system.

Assays based on colloidal AuNPs allow a naked-eye visualization of the results because AuNPs color depends on their size, shape, and inter-particle distance^24,25^. AuNPs produced by the citrate reduction method were stabilized in solution of wine-red color (Fig. 3A). TEM observations showed that the AuNP morphology was uniform and spherical of about 20 nm diameter (Fig. 3B). Applying the multipole scattering theory on UV–Vis spectrum of synthetized AuNPs^26^ (Supplementary Fig. S2) the calculated particle size was 22.1 nm and their concentration 3.7 × 10^12^ NPs/mL (Supplementary Table S1). Moreover, particles had a quite narrow size distribution with a hydrodynamic diameter of about 20 ± 3 nm, as estimated from DLS measurements (Fig. 3C). Obtained AuNPs were stable at 4 °C for at least six months. The addition of 5 mM MgCl_2_ induced the color change of the solution to violet (Fig. 3A), due to the ionic strength-induced cutoff of electrostatic repulsive forces between nanoparticles causing their aggregation. AuNPs aggregation was confirmed by DLS measurements because the hydrodynamic diameter shifted to 170 ± 5 nm (Fig. 3C). Naked-eye observation of AuNPs was possible due to the phenomenon of local surface plasmon resonance^27^. The initial solution of a red color had plasmonic band at ∼520 nm, while the solution with aggregated AuNPs of a blue-to-violet color had plasmonic band at ∼630 nm (Fig. 3D).

### Colorimetric detection of LAMP products

The principle of the LAMP-AuNPs assay is described in Fig. 3E. Initially, the LAMP products were incubated with colloidal AuNPs to enable their interaction. Oligonucleotides have a strong tendency to stably adsorb on the surface of AuNPs through electrostatic interactions, as previously described in reference^25^. Since the adsorption of amplicons on AuNPs protects nanoparticles from salt-induced aggregation, the addition of MgCl_2_ induced no color change in solution containing LAMP products. However, the color of AuNPs incubated with negative LAMP reaction, containing no amplicons, was sensitive to MgCl_2_ addition and became violet.

**Figure 4:**
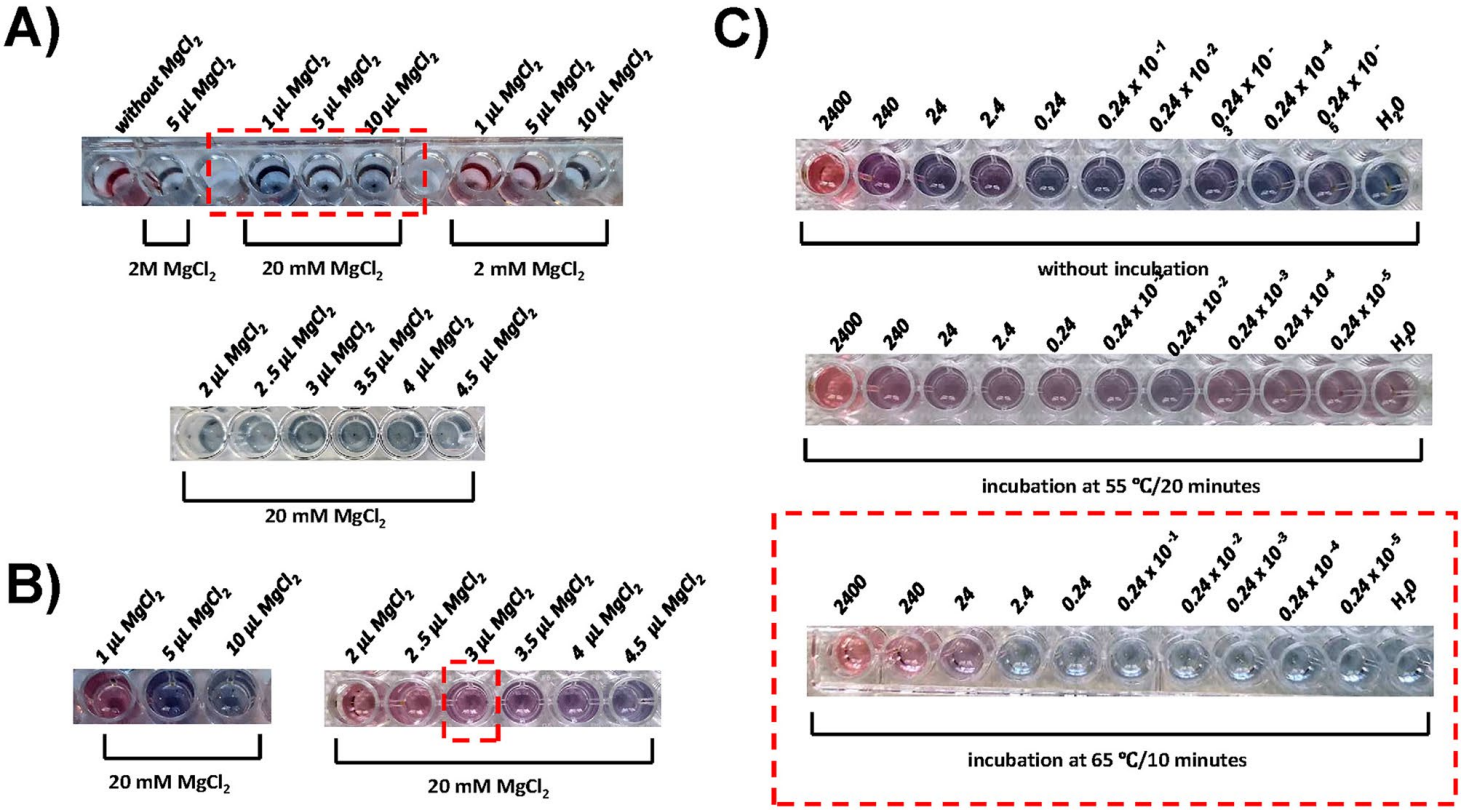
Optimization of colorimetric detection of LAMP products. (**A**) Impact of salt concentration on the aggregation of AuNPs. (**B**) Impact of salt concentration on the aggregation of AuNPs hybridized with LAMP amplicons. (**C**) Optimization of incubation time and temperature to allow AuNP hybridization with LAMP amplicons. The numbers stand for concentrations of LAMP amplicons in ng/μL. Circled in red are chosen conditions.

For the sensitive detection of LAMP products, AuNPs should be aggregated properly in response to a very small amount of salt. In a high salt condition, citrate-capped AuNPs readily aggregate and the number of amplicon molecules needed to suppress the aggregation is high. Therefore, to enhance the sensitivity of the colorimetric detection, we first optimized the concentration of MgCl_2_. The impact of salt concentration on the aggregation of AuNP was tested in 150 µL volume containing 2 × 10^9^ NPs. This volume was chosen in order to adapt the assay to a multiplex 96-well microplate format. When 1, 5, or 10 μL of MgCl_2_ of varying molarities (2 mM, 20 mM, and 2 M) were added to the AuNP solutions, the agglomeration of gold nanoparticles was observed across all tested concentrations obtained with 20 mM MgCl_2_ stock solution i.e., from 0.13 mM to 1.33 mM final concentration (Fig. 4A). We, thus, proceeded to utilize 20 mM MgCl_2_ as a salt stock solution in subsequent tests with LAMP products. When AuNPs were preincubated with 2 µL of LAMP products (32 ng/µL in final concentration), 3 min after the addition of up to 0.4 mM MgCl_2_, the solution retained a red color, indicating the prevention of salt-induced aggregation due to the adsorption of amplicons onto AuNPs (Fig. 4B). Therefore 0.4 mM MgCl_2_ was chosen for the colorimetric sensor construction.

Next, various temperatures and time conditions were tested to improve binding of the LAMP products to gold nanoparticles. The corresponding results, depicted in Fig. 4C, indicate that incubation at 65 °C for 10 min was the most optimal for the adsorption of LAMP products with AuNPs and was used in further experiments.

**Figure 5:**
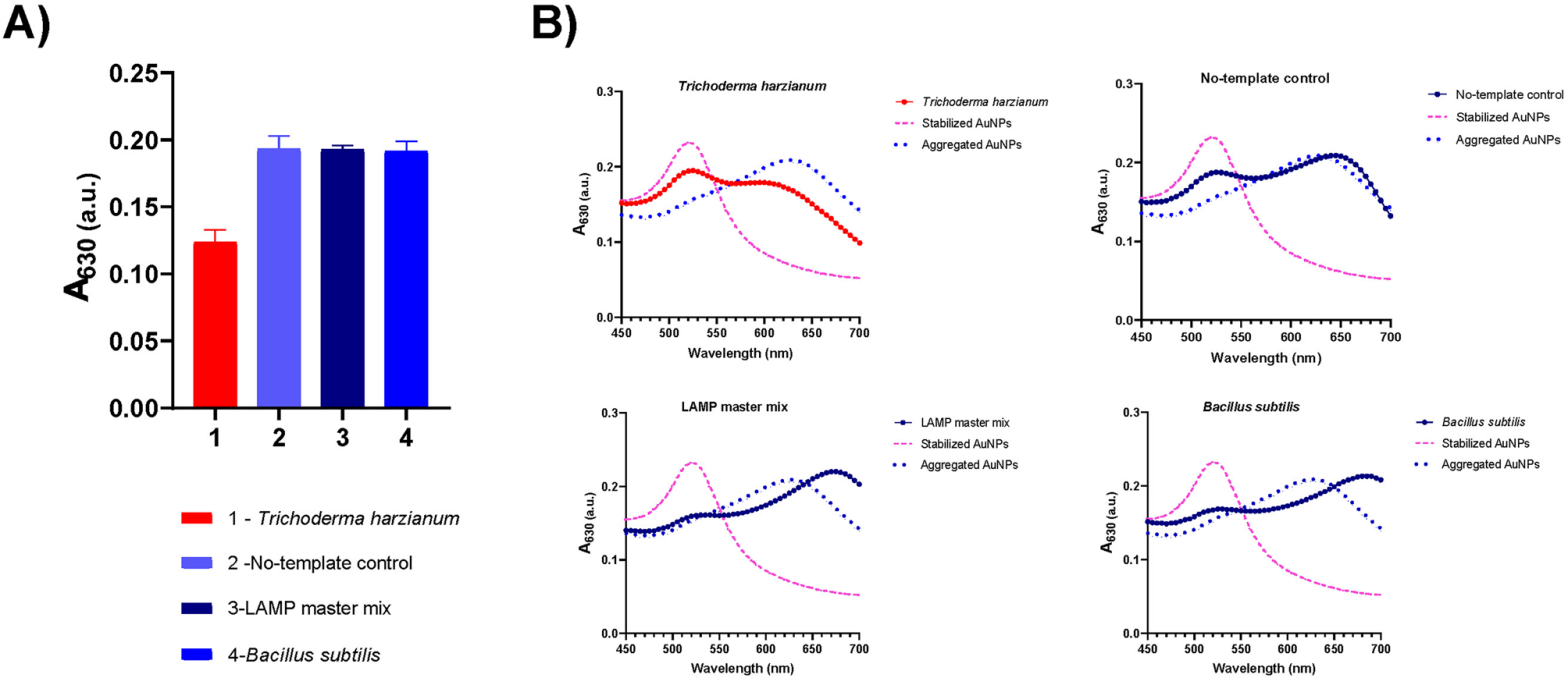
Detection of LAMP products. (**A**) Absorption at 630 nm (A_630_) for the detection of the positive LAMP reaction and negative controls. (**B**) Absorbance spectra of the positive reaction and tested control coupled with AuNPs. Dashed lines present citrate-capped AuNPs (red) and salt-aggregated AuNPs (blue).

To assess the specificity of the LAMP-AuNP assay, AuNPs were incubated with a positive LAMP reaction obtained using gDNA of *T. harzianum* isolated from substrate used for *A. bisporus* cultivation. Negative LAMP reactions were performed without DNA template, or with non-specific gDNA from *B. subtilis* together with LAMP master mix (WarmStart LAMP Reagent containing deoxynucleotides dNTPs, *Bst* 2.0 WarmStart DNA Polymerase, isothermal amplification buffer, LAMP primers and PCR-grade water). As shown in Fig. 5, the color of solutions and spectrophotometric measurements indicated that only AuNPs incubated with positive LAMP were not aggregated upon MgCl_2_ addition. These results indicate that the color remained red in a specific way as a result of the binding between LAMP amplicons and AuNPs.

**Figure 6:**
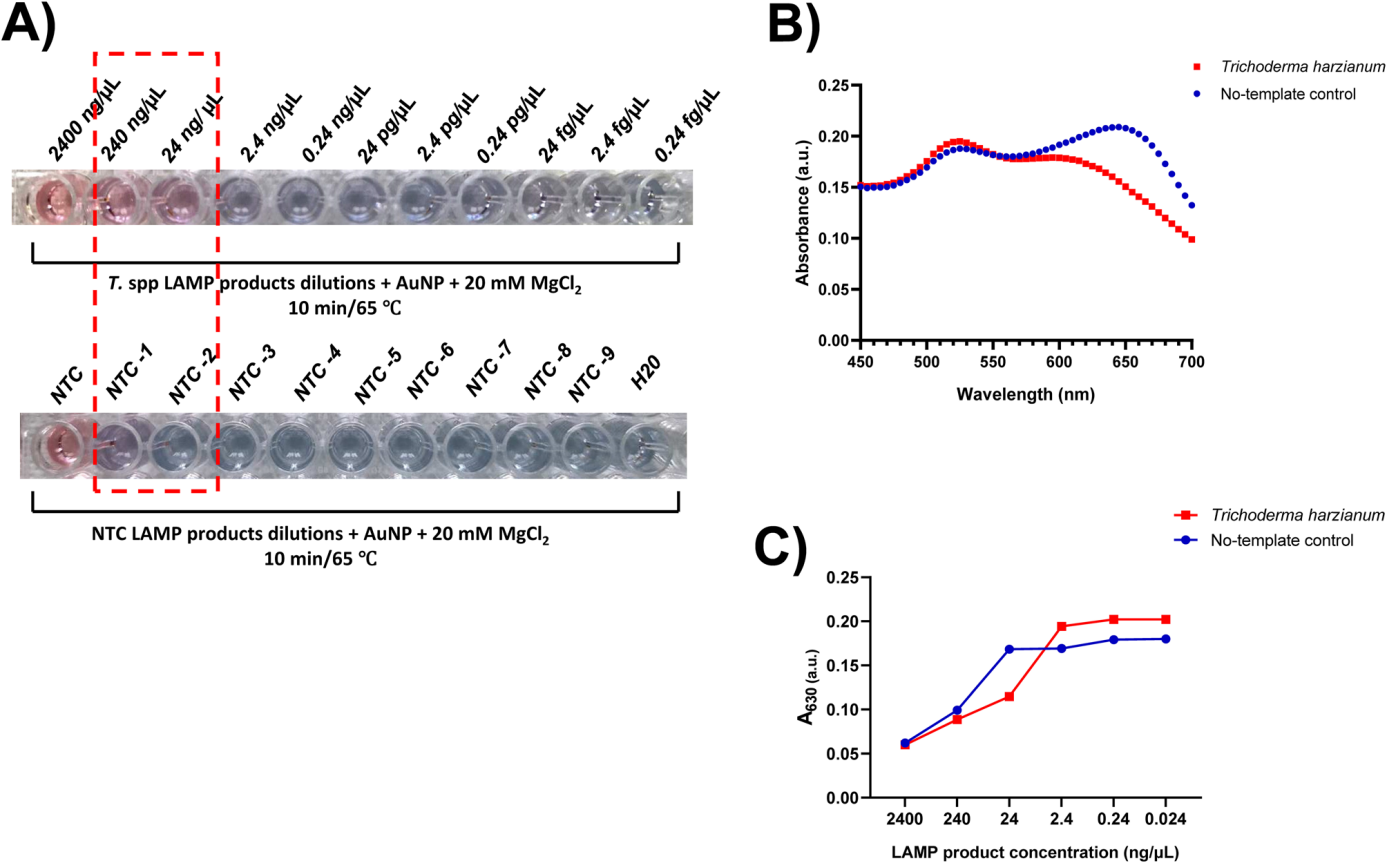
Sensitivity of the LAMP-AuNP assays. (**A**) Color differences in positive (above) and negative (below) LAMP reactions detection using the colorimetric test at different dilutions. Circled in red are dilutions providing discriminations between positive and negative LAMP reactions. (**B**) The absorbance spectra of the AuNPs assay performed with two-time dilution of positive (red) and negative (blue) LAMP reaction; (**C**) The absorbance intensity at 630 nm (A_630_) of various dilutions of positive (red) and negative (blue) LAMP-AuNP assays.

To determine the minimum detectable amount of the LAMP product by the AuNPs-based assay, tests were carried out with tenfold serial dilutions of positive LAMP reaction products obtained with *T. harzianum* gDNA. In parallel, serial dilutions of the no-template reaction and negative LAMP reaction with non-specific gDNA of *B. subtilis* were also tested. In case of the positive LAMP reaction a limit of detection of 24 ng/µL was observed by naked eye (Fig. 6A). At this amplicon dilution there was a clear difference in absorption intensity at 630 nm between the positive LAMP reaction and all tested negative controls (Fig. 6B). The comparison of signal intensities at A_630_ showed that the distinction between positive and negative LAMP reactions is not possible directly in crude samples but requires a ten-time dilution. This can be explained by the high concentration of reactants and primers in the LAMP reaction and master mix that prevent salt-induced AuNPs aggregation. In diluted samples the concentration of reagents and primers decreased and AuNPs aggregation is controlled only by the presence of amplicons. Figure 6C indicates that the LAMP-AuNPs assay was sensitive in solution containing amplicons at concentrations ranging from 240 to 24 ng/µL. Further dilution of samples overly decreased the number of amplicons and no suppression of AuNP aggregation was possible at the given salt concentration.

Overall, to observe the color change from red (indicating a positive result—the presence of *Trichoderma*) to blue (indicating a negative reaction—the absence of *Trichoderma*) using the LAMP-AuNPs assay, it is necessary to test several dilutions at the same time, for which the 96-well plate was found to be suitable. It is also necessary to strictly follow good laboratory practice in order to avoid potential cross-contamination and/or false-positives. On this note, it is important to add that LAMP has been known for producing false-positives, however it has been shown that the longer the duration of reaction, the higher proportion of false-positives, and the earliest false-positives appeared after 45 min^28^. Therefore, the time-gating strategy we used (the assay duration is 30 min) should prevent the formation of false-positives. Further optimization may be needed to enable a proper translation of the developed LAMP-based assay to the fully POC-applicable kit.

In summary, to the best of our knowledge, there is currently no other publication describing LAMP-based assay for detection of *Trichoderma* on a genus-specific level, performed using the newly designed and validated LAMP primers, label-free AuNPs and direct visualization of the positive LAMP reaction, yielding detection sensitivity by LAMP of at least 917 fg/µL (Supplementary Fig. S7).

## Conclusions

In this work, we combined the merit of LAMP and colorimetric monitoring of amplicons using AuNPs in a high-throughput format to detect *Trichoderma*. We demonstrated the efficiency of the assay on the soil-borne *T. harzianum* species isolated from substrates from organic button mushroom farming. The test shows potential for being routinely performed for screening mushroom substrates (compost and casing soil). However, since the test is based on salt-induced AuNPs aggregation and localized surface plasmon phenomenon, its working conditions depend strongly on the size and shape of used AuNPs^25,29–33^. Changing the type of AuNPs will demand a new optimization of salt-concentration and time of reaction to enable naked-eye detection. The colorimetric signal provides a simple and quick result acquisition independent of the number of samples present on the plate.

The portability and naked-eye visualization of results may help future development of a high-throughput sensitive *Trichoderma* spp. detection directly at the farm. The test can be easily utilized as a smartphone-based colorimetric test since many phone applications are available that can image and analyze 96 well plates (such as e.g. free app MyLight). This assay represents a good alternative to other analytical methods coupled with LAMP and reports an innovative concept for *Trichoderma* spp. detection.

## Materials and methods

### Reagents and kits

Sodium citrate, gold (III) chloride solution (HAuCl_4_), GelRed Nucleic Acid Stain, and magnesium chloride (MgCl_2_) were purchased from Sigma-Aldrich (Saint-Quentin-Fallavier, France). Hydrogen chloride (HCl) and nitric acid (HNO_3_) was purchased from VWR (Strasbourg, France) and Merck (Saint-Quentin-Fallavier, France), respectively. LAMP primers were synthetized by Integrated DNA Technologies (Coralville, Iowa, USA) and resuspended in MilliQ water. Malt Agar was obtained from Torlak Institute of Immunology and Virology (Belgrade, Serbia), Rose Bengal Agar (RBA) was purchased from Merck (Darmstadt, Germany), and Tryptone Soya Agar (TSA) from Millipore (Burlington, USA).

The DNeasy Blood & Tissue Kit was purchased from Qiagen (Düsseldorf, Germany). Plant/Fungi DNA Isolation Kit was obtained from Norgen Biotek (Thorold, Canada), and GenElute™ Soil DNA Isolation Kit was purchased from Sigma-Aldrich (LLC, Germany). WarmStart LAMP Kit (DNA & RNA) was purchased from New England Biolabs (Massachusetts, USA).

### Microorganisms and DNA extraction

The soil-borne *T. harzianum* was isolated through cultivation and co-cultivation process from the button mushroom substrate samples (compost/casing soil) kindly provided by a local organic mushroom producer in Serbia. Samples of compost and casing soil, from which a pure culture of *T. harzianum* was isolated, were collected from 10 cultivation bags at the conclusion of the button mushroom cultivation process. At this stage the highest concentration of *Trichoderma* spores in the substrate was expected. Samples for *Trichoderma* isolation were cultivated on the same day. After cultivation and co-cultivation, pure isolates were obtained. Initially, cultures were grown on RBA at 26 °C for 5 to 7 days. Subsequently, fungal cultures which have grown from the samples were transferred to Malt Agar and allowed to grow at 26 °C for another 5–7 days to obtain pure cultures. *T. harzianum* was identified by sequencing (Novogene sequencing services) after DNA extraction from the pure cultures using GenElute™ Soil DNA Isolation Kit. *Aspergillus fumigatus*, *Cladosporium allicinum*, and *Alternaria alternata* were used as negative controls (Table 1). *B. subtilis*, used as a soil bacterial control strain, was cultivated in TSA at 37 °C overnight. All species and the strain used in this study are given in Table 1 and can be accessed using the following link: https://zenodo.org/records/11385670 (10.5281/zenodo.11385670).

**Table 1:** Fungal species and bacterial strains used in this study.

Species/strain	Source/collection	ID number
*Trichoderma harzianum*	Biosense culture collection of fungi^a^	BSCCF-002
*Aspergillus fumigatus*	Biosense culture collection of fungi^a^	BSCCF-005
*Alternaria alternata*	Biosense culture collection of fungi	BSCCF-006
*Cladosporium allicinum*	Biosense culture collection of fungi	BSCCF-007
*Bacillus subtilis*	Biosense culture collection of bacteria^b^	BSCCB-007

^a^This work; ^b^Ref^34^.

The extraction of genomic DNA (gDNA) from fungal cultures and *B. subtilis* was conducted by using Plant/Fungi DNA Isolation Kit and DNeasy Blood & Tissue Kit, respectively, following the manufacturer’s protocol. DNA concentration and purity were measured using a BioSpec-nano spectrophotometer (Shimadzu, Kyoto, Japan). The extracted DNA samples were stored at −20 °C until further use.

### AuNPs production and characterization

The AuNPs were synthesized using the reduced citrate method as described previously^27^. Briefly, the synthesis was performed in an ultraclean 250 mL Erlenmeyer flask glassware washed in aqua regia (HCl/HNO_3_ 3:1, v/v), rinsed three times in MilliQ water and dried to prevent undesired nucleation and gold colloid aggregation. Stock solutions of 50 mM HAuCl_4_ and 38.7 mM of sodium citrate were prepared in MilliQ water. 500 µL of the gold solution was added to 125 mL MilliQ water and vigorously stirred and heated to 95 °C. Then, 2 mL of the sodium citrate solution was added to the solution, resulting in a rapid color change from pale yellow to dark red. After few minutes, the solution slowly turned to red-wine color. After cooling, the resulting AuNPs were kept at 4 °C. Absorbance measurements were performed using an UV–Vis spectrometer (Agilent Technologies, Santa Clara, CA) and quartz cuvette of 1 cm path length in the wavelength range from 200 to 800 nm (with the wavelength increment of 5 nm) in order to estimate the AuNPs concentration. Transmission electron microscopy (TEM) was used to capture images of the nanoparticles (CM120 TEM, Philips, Amsterdam, Netherlands) with an accelerating voltage of 120 kV at the Centre Technologique des Microstructures-Lyon 1, Villeurbanne, France. AuNPs were examined after deposition of 5 µL of diluted solutions on a formvar-coated copper grid (Electron Microscopy Sciences, PA, USA) and evaporation to dryness. The hydrodynamic diameter of AuNPs was estimated using dynamic light scattering (DLS) measurements (Zetasizer Pro, Malvern Panalytical, Paleaseau, France).

### LAMP assay

LAMP assays were performed targeting the *tef1* gene of *T. harzianum*, which is known to encode translation elongation factor 1-alpha. The LAMP primers were designed using the Primer Explorer V5 software (http://primerexplorer.jp/lampv5e/, accessed on 10 January 2023) (Eiken Chemical Co., Ltd) and a 1280-bp sequence of the *tef1* gene (GenBank accession no. OL435125). The LAMP primer set comprised six primers, including two outer primers (forward and backward outer primer, F3 and B3, respectively), two inner primers (forward and backward inner primer, FIP and BIP, respectively), and two loop primers (forward and backward loop primer, LF and LB, respectively). Sequences of the primers are shown in Table 2. Reactions consisted of 1 μL of target gDNA sample in 25 μl of master mix composed of 0.2 μM outer primers (F3, B3), 1.6 μM inner primers (FIP, BIP), 0.4 μM loop primers (LF, LB), 12.5 µL of the WarmStart LAMP Kit (DNA & RNA) (New England Biolabs), 0.5 µL of fluorescent dye (50X) and 8.5 µL of PCR-grade water. The preparation of all LAMP reactions was carried out following good laboratory practices, such as sterilizing the workspace before and after experiments, using aliquoted reagents and primers, sterilizing gloves with ethanol between pipetting each sample, and pipetting the reagents first, while adding the DNA last to avoid contamination of the LAMP kit and primers. The reactions were performed on a Genie^®^ III (OptiGene, UK), an instrument for isothermal nucleic acid amplification, by varying the reaction temperature (60 °C, 62 °C, 65 °C) and time of reaction (30 min and 60 min) in order to optimize conditions (Supplementary Figs. S3–S6). For gel electrophoresis, 2% agarose gel was prepared in 1× TBE buffer (Tris–borate-EDTA) and stained with 3 µg/mL (3×) of GelRed. Then, 5.0 μL of LAMP amplicons mixed with 1.0 μL of the loading dye (New England Biolabs) were loaded into the gel wells. The voltage was set to 140 V and started. The assay lasted 45 min. The results on the gel of 10.5 × 8.3 cm were observed by the proBLUEVIEW Dual Colour transilluminator (Cleaver Scientific, Rugby, United Kingdom).

**Table 2:** LAMP primers for specific detection of *Trichoderma* spp.

	Primer name	Sequence 5ʹ–3ʹ	Position	Length
LAMP primers specific for *Trichoderma* spp.	Trich-F3	GTATGGTCGTCACCTTCGC	1027–1045	19
	Trich-B3	GGGTGGTTCATGACGATGAC	1233–1252	20
	Trich-FIP (F1c + F2)	AACACCCTCGACGAGCTGCT-CCTCCAACGTCACCACTGA	N.A	39
	Trich-BIP (B1c + B2)	AATTCGCCGTGGTAACGTTGCC-GAAAGAAGCGGCACCCAT	N.A	40
	Trich-LF	GGTGCATCTCGACGGACTT	1071–1089	19
	Trich-LB	TGACTCCAAGAACGACCCC	1182–1201	19

### Colorimetric detection of LAMP products

2 µL of the obtained LAMP amplicons were carefully transferred to the well containing 50 µL of AuNPs and 98 µL of MilliQ water following good laboratory practices (one by one tube) to avoid potential cross-contamination. The mixture was then incubated at 65 °C for 10 min. Subsequently, 2.5 µL of 20 mM MgCl_2_ was added and the color change was observed by naked-eye or by measuring the absorption of solution using the SPARK multimode microplate reader (Tecan Global Headquarters, Männedorf, Switzerland).

To determine the minimum detectable amount of the LAMP product by AuNPs, an investigation was carried out involving a tenfold serial dilution of positive LAMP reaction products in the context of *T. harzianum* gDNA detection. Ten-fold serial dilutions were executed using sterile deionized MilliQ water, ranging from dilutions at 2400 ng/μL to 2.4 fg/μL.

### Supplementary Information

Supplementary Information.

## Data Availability

All data generated or analyzed during this study are included in this published article (and its supplementary information files).
