# Supplementary material for: Loop-mediated isothermal amplification (LAMP) assay coupled with gold nanoparticles for colorimetric detection of Trichoderma spp. in Agaricus bisporus cultivation substrates
Source: Sci Rep. 2024 Jul 5;14:15539. doi: 10.1038/s41598-024-65971-9 (PMC11226595; doi:10.1038/s41598-024-65971-9)
Supplement: Supplementary file 1 — Supplementary Information. [file 41598_2024_65971_MOESM1_ESM.docx]

***Supplementary data***

**Loop-Mediated Isothermal Amplification (LAMP) assay coupled with Gold Nanoparticles for Colorimetric Detection of *Trichoderma* spp. in *Agaricus bisporus* Cultivation Substrates**

# Mila Djisalov^1*^, Ljiljana Janjušević^1^, Vincent Léguillier^2^, Ljiljana Šašić Zorić^1^, Carole Farre^3^, Jamila Anba-Mondoloni^2^, Jasmina Vidic^2*^, Ivana Gadjanski^1*^

^1^ University of Novi Sad, BioSense Institute, Novi Sad, Serbia
^2^ Université Paris-Saclay, Micalis Institute, INRAE, AgroParisTech, UMR 1319, Jouy en Josas, France
^3^ Université Claude-Bernard Lyon 1, CNRS, Institute of Analytical Science, 69100 Villeurbanne, France
[jasmina.vidic@inrae.fr](mailto:jasmina.vidic@inrae.fr); [igadjanski@biosense.rs](mailto:igadjanski@biosense.rs)

**Contact information for Corresponding authors**

J.V. [jasmina.vidic@inrae.fr](mailto:jasmina.vidic@inrae.fr); I.G. [igadjanski@biosense.rs](mailto:igadjanski@biosense.rs) and M.Dj [mila.djisalov@biosense.rs](mailto:mila.djisalov@biosense.rs)

**SUMMARY**

**Supplementary Data S1.** Alignment – LAMP primer specificity for the chosen target *- GenBank* gene for potential cross-reaction with other fungal species

**Supplementary Data S2.** Alignment – LAMP primer specificity confirmation with the *GenBank* genes of a wide variety of *Trichoderma* spp.

**Supplementary Figure S1.** The position and direction of all LAMP primers within the  *GenBank* gene **Supplementary Figure S2**. Absorbance spectrum of synthetized AuNPs **Supplementary Table S1.** AuNPs characterization **Supplementary Figure S3.** LAMP optimization – testing the reaction at 60 ℃ during 60 minutes **Supplementary Figure S4.** LAMP optimization – testing the reaction at 62 ℃ during 60 minutes **Supplementary Figure S5.** LAMP optimization – testing the reaction at 65 ℃ during 60 minutes **Supplementary Figure S6.** LAMP optimization – testing the reaction during 30 minutes **Supplementary Figure S7.** LAMP assay sensitivity testing **Supplementary Figure S8.** An image of the full-size, uncropped 2% agarose gel

**References**


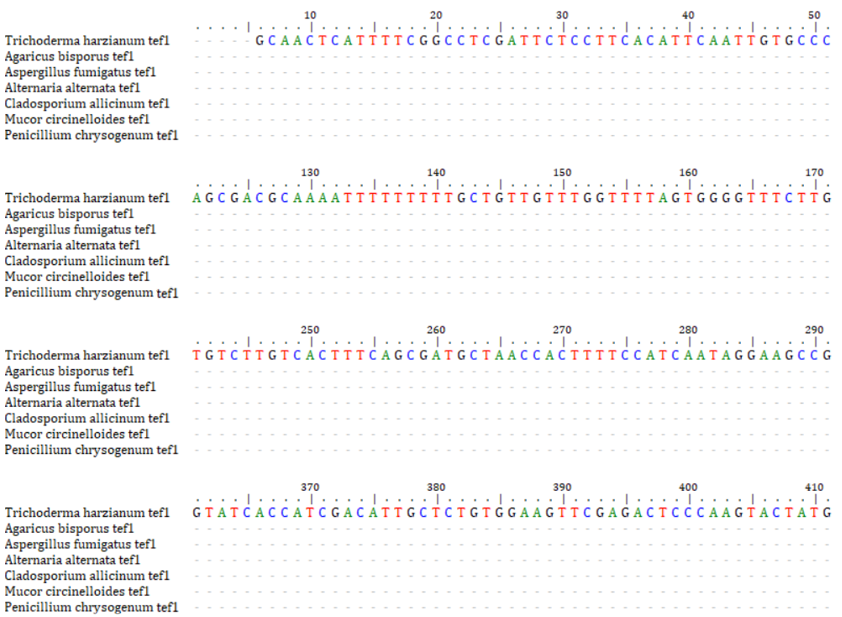


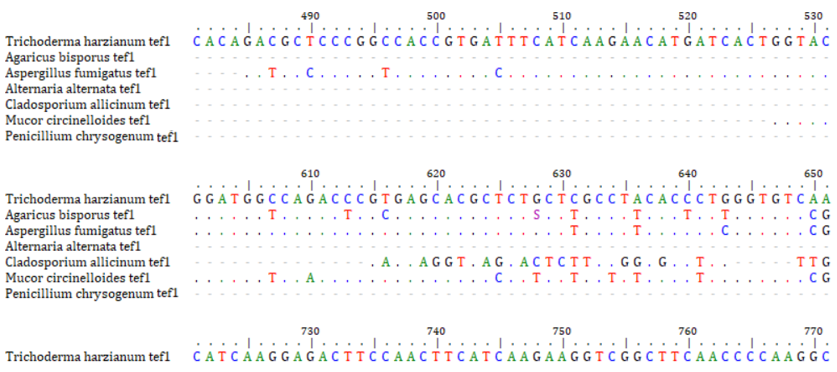


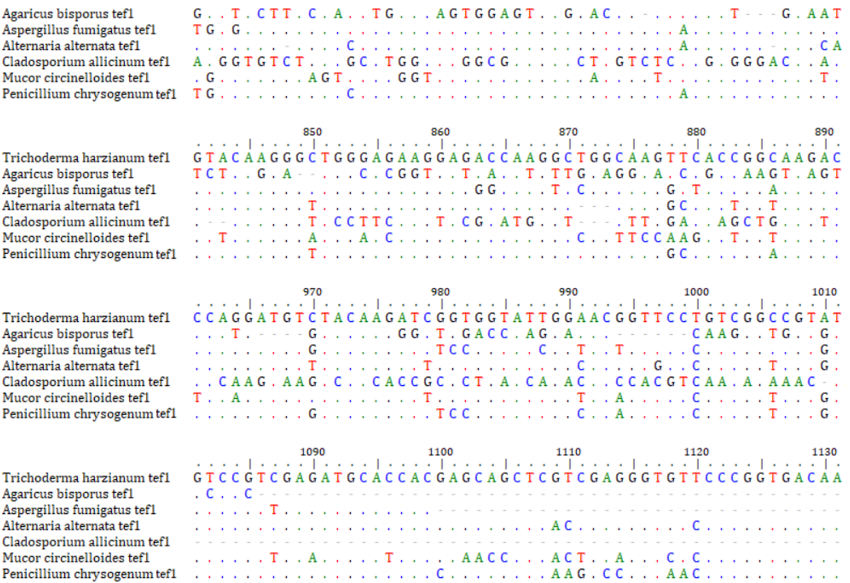


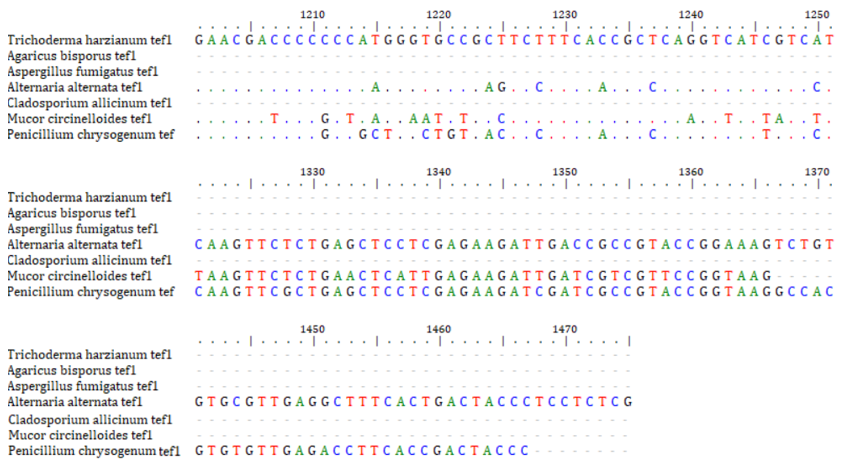


**Supplementary Data S1**. Sequence alignment of *tef1* gene done in BioEdit v7.2.0 software (Informer Technologies, Inc.) to check for sequence similarity between *Trichoderma harzianum* (GenBank: OL435125) and different fungal species known to be widely represented in the soil materials such as compost and casing soil (*Agaricus bisporus* – GenBank: KR006640, *Aspergillus fumigatus* - GenBank: KM921968.1, *Alternaria alternata* - GenBank: MN970210, *Cladosporium allicinum* - GenBank: OK509507, *Mucor circinelloides* - GenBank: LT220806, *Penicillium chrysogenum* - GenBank: KJ476405).


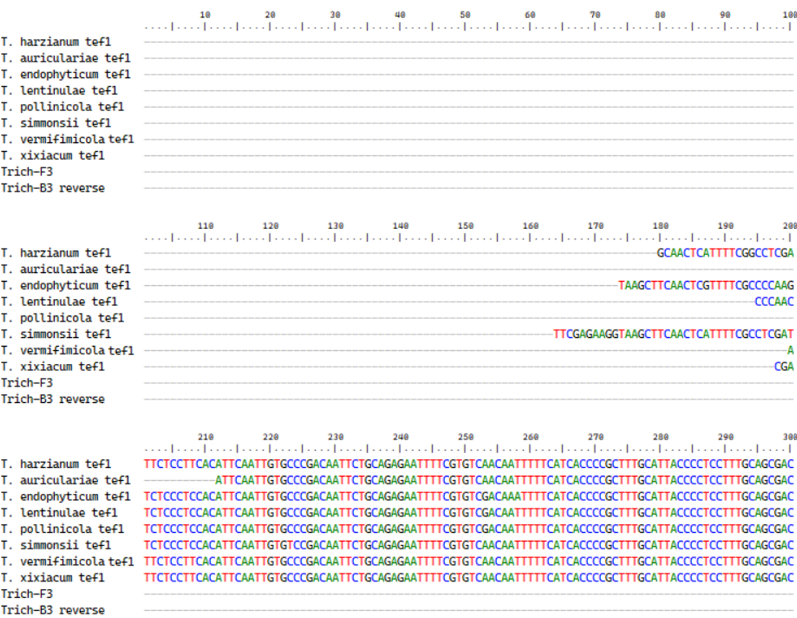


**a)**

**
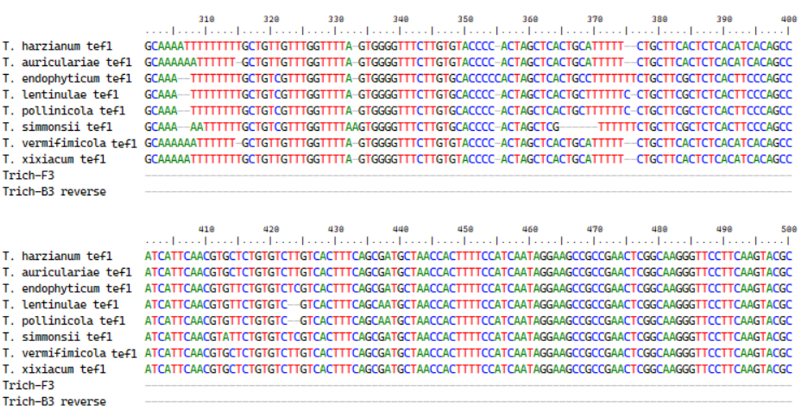
**

**
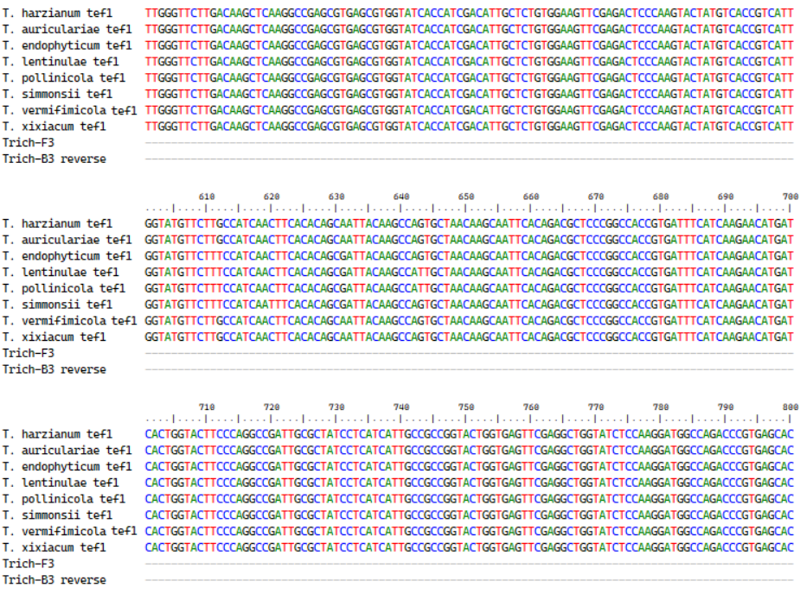
**

**
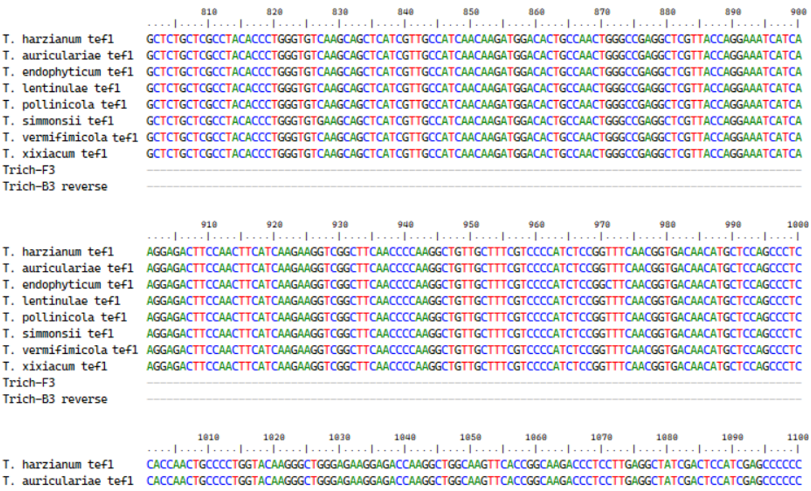
**

**
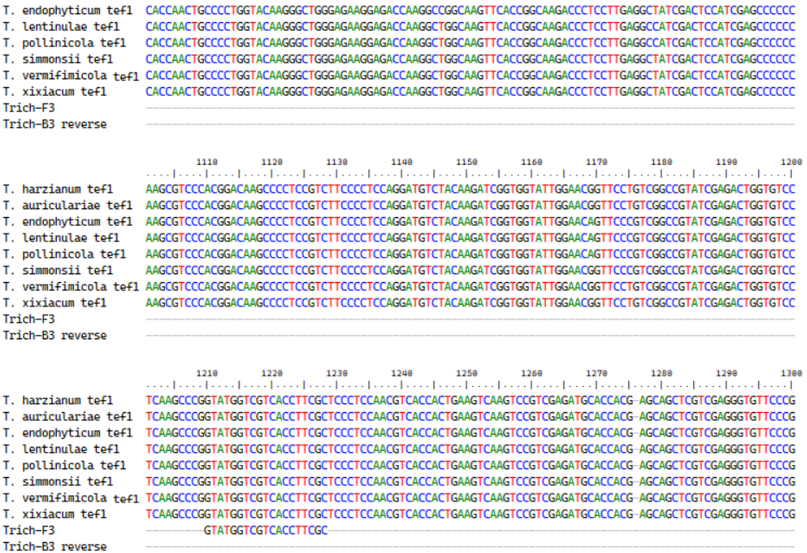
**

**
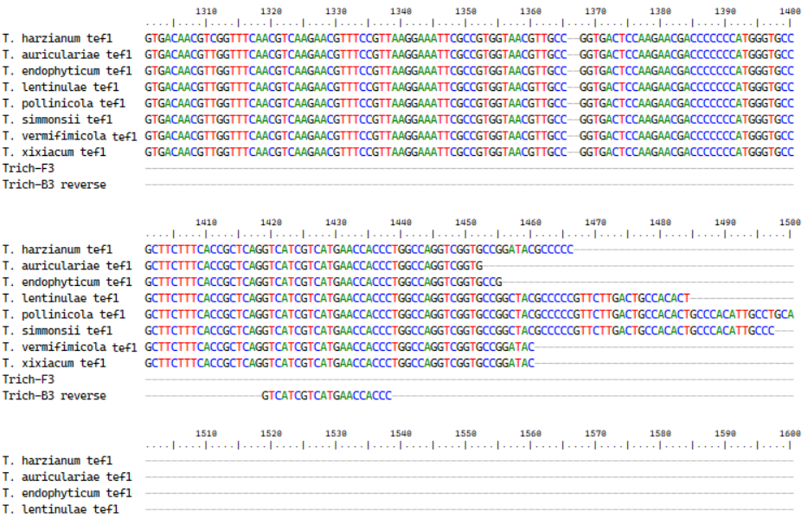
**

**
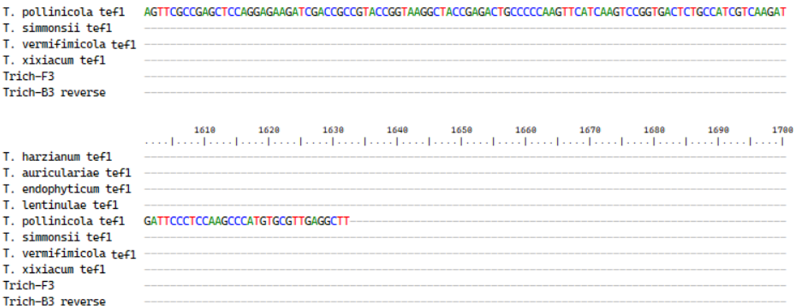
**

**b)**

**
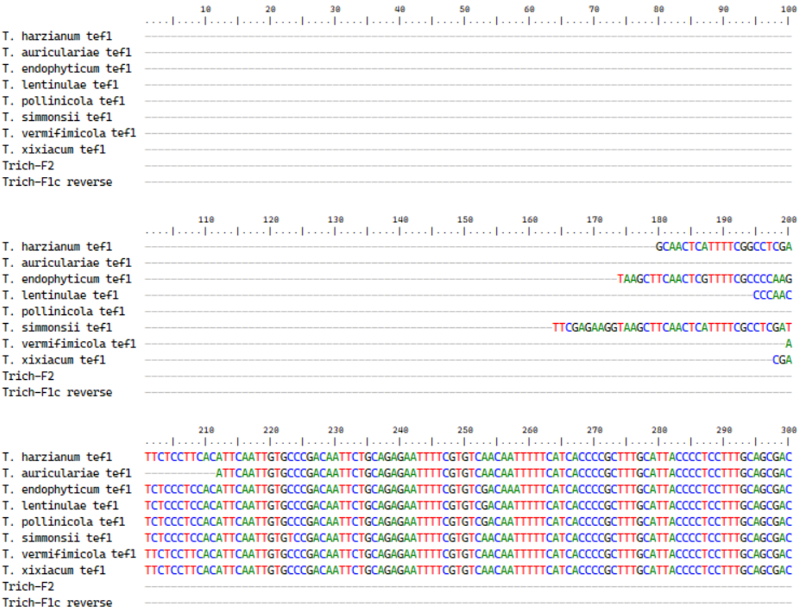
**

**
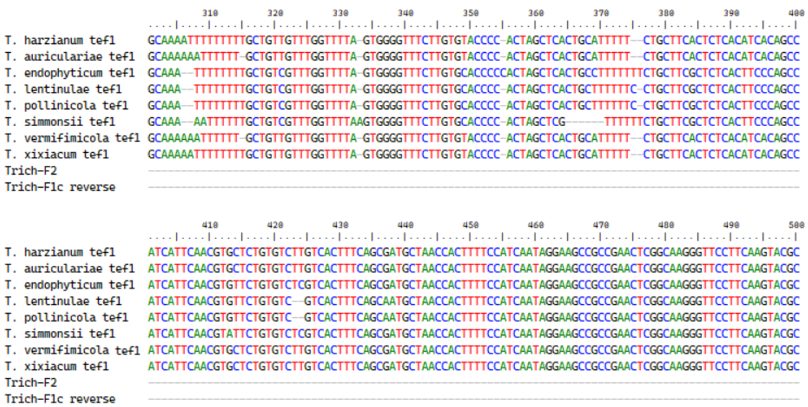
**

**
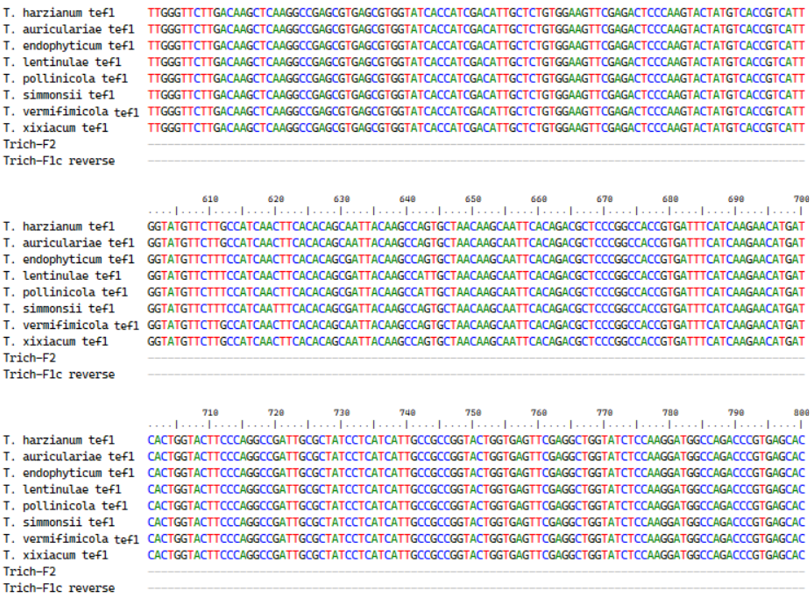
**

**
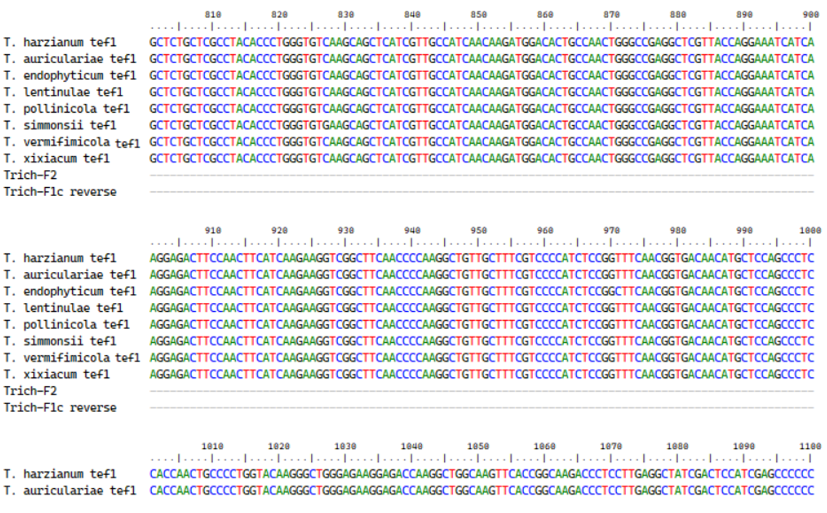
**

**
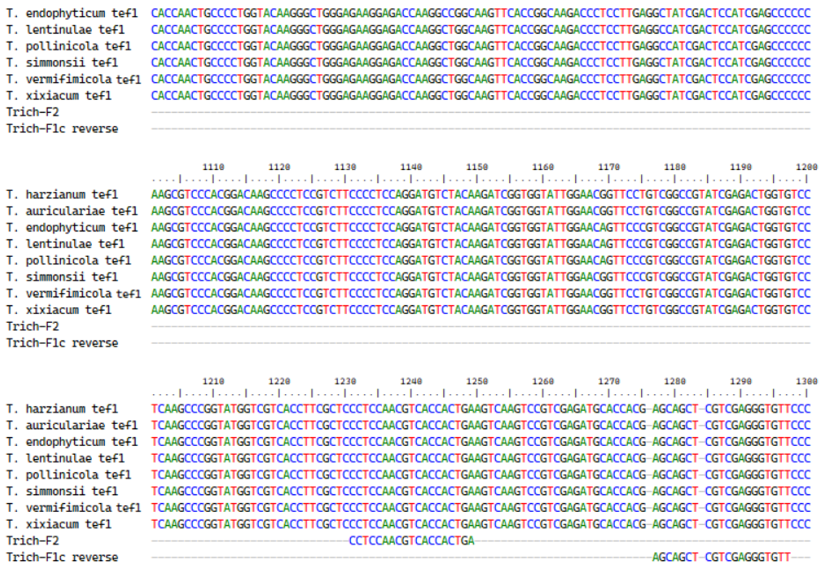
**

**
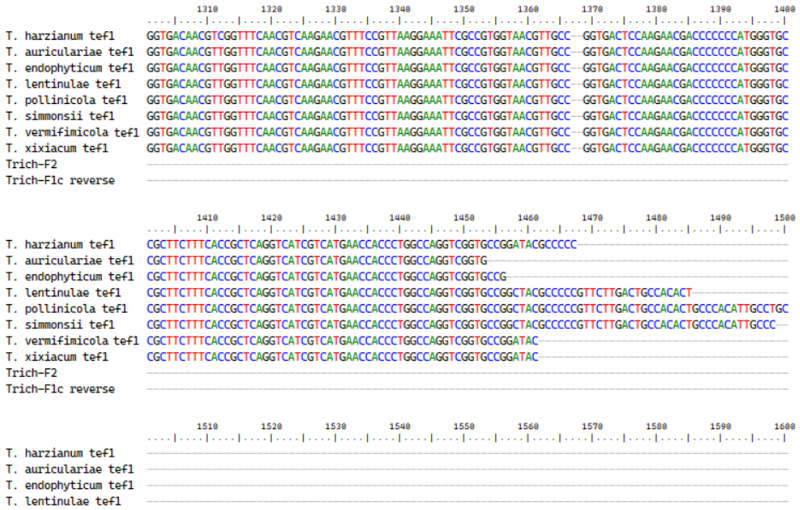
**

**
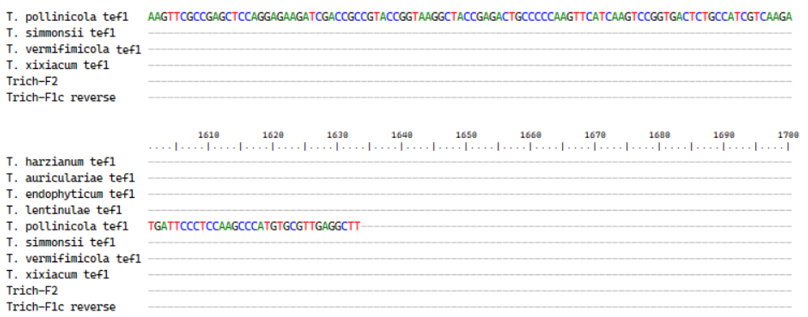
**

**
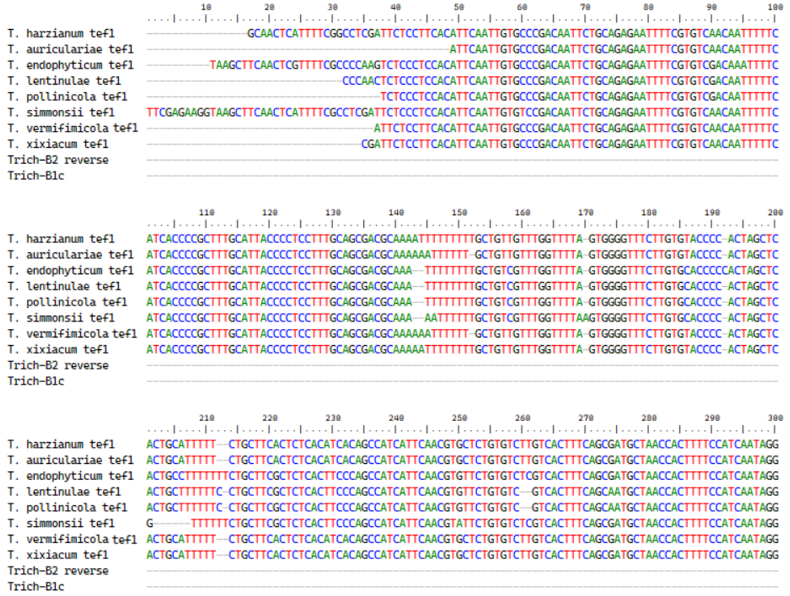
**

**c)**

**
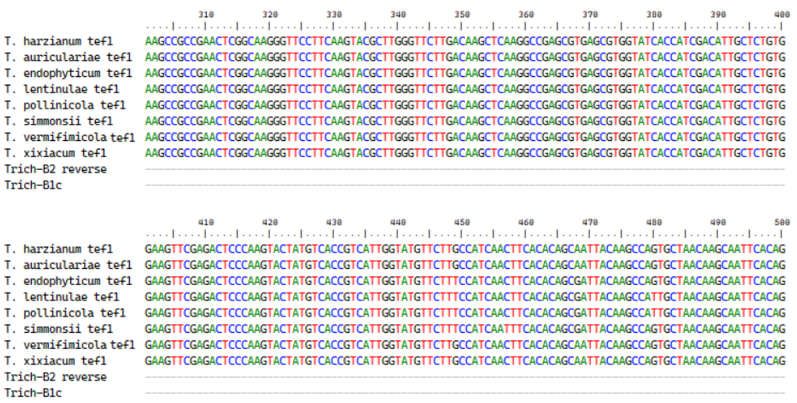
**

**
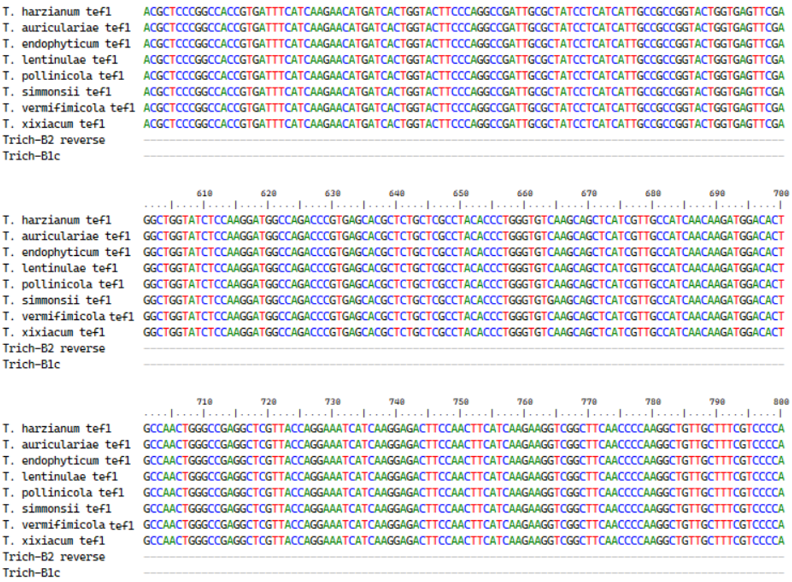
**

**
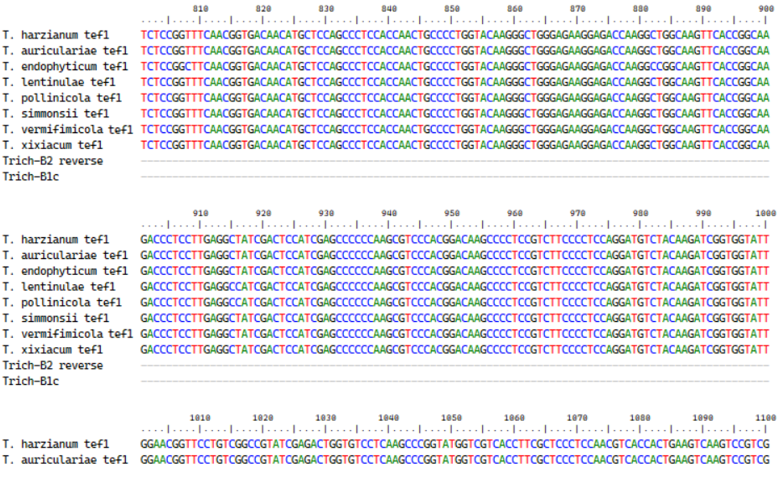
**

**
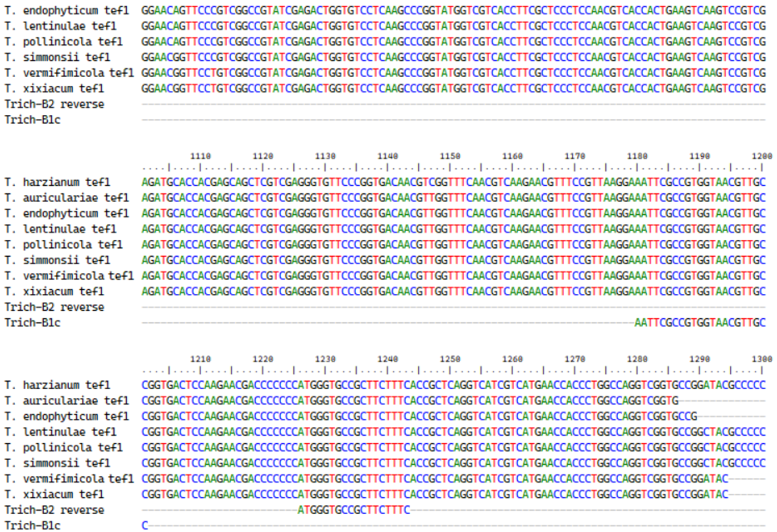
**

**
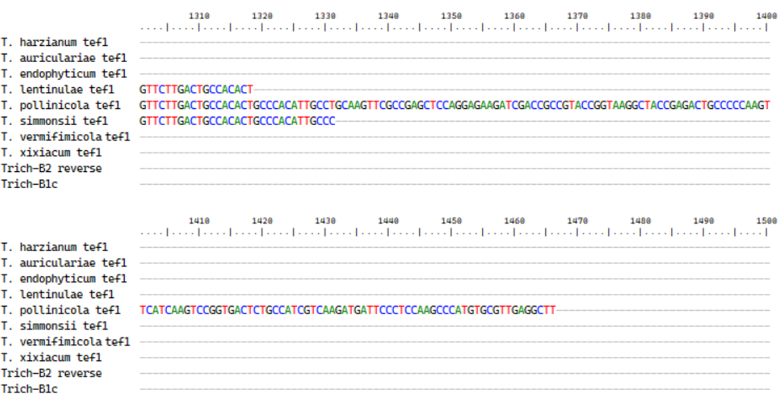
**

**
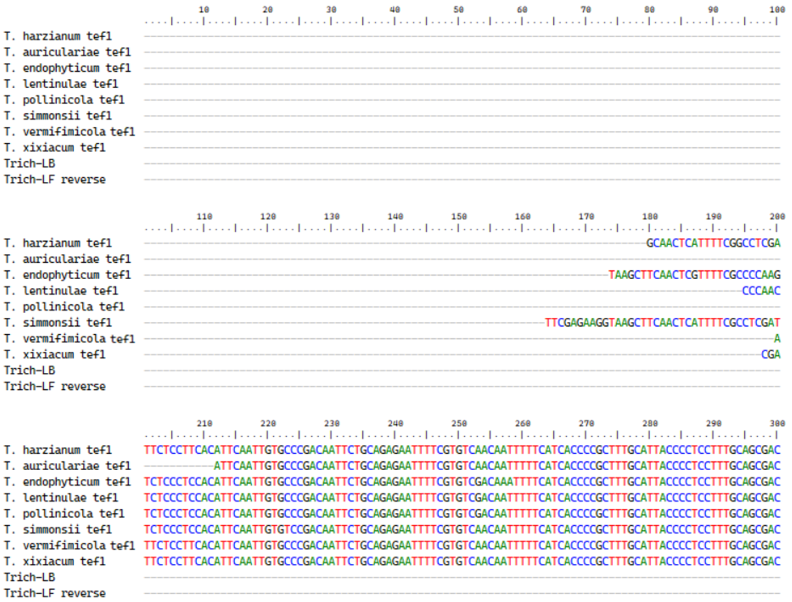
**

**d)**

**
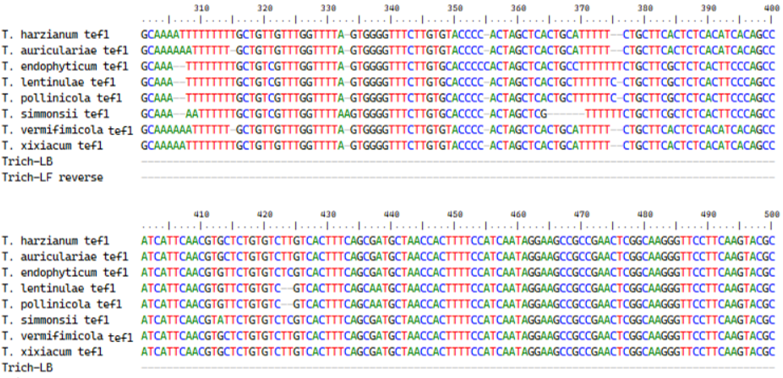
**

**
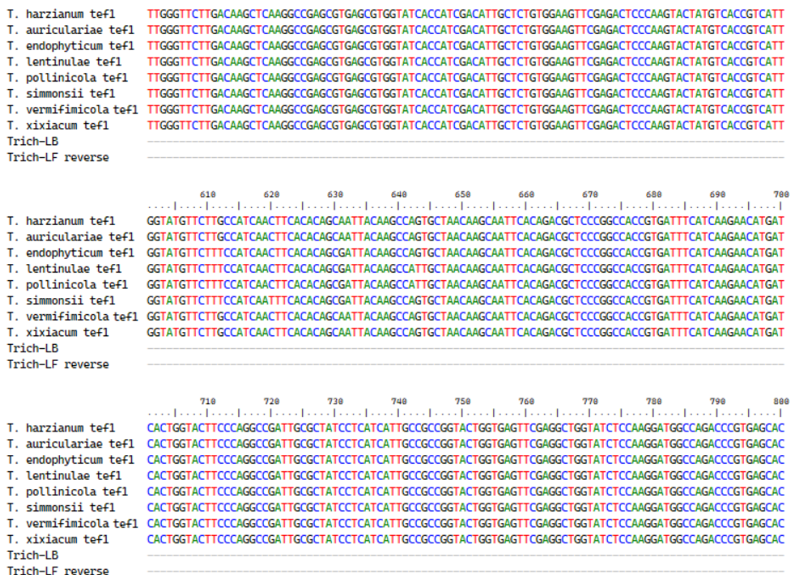
**

**
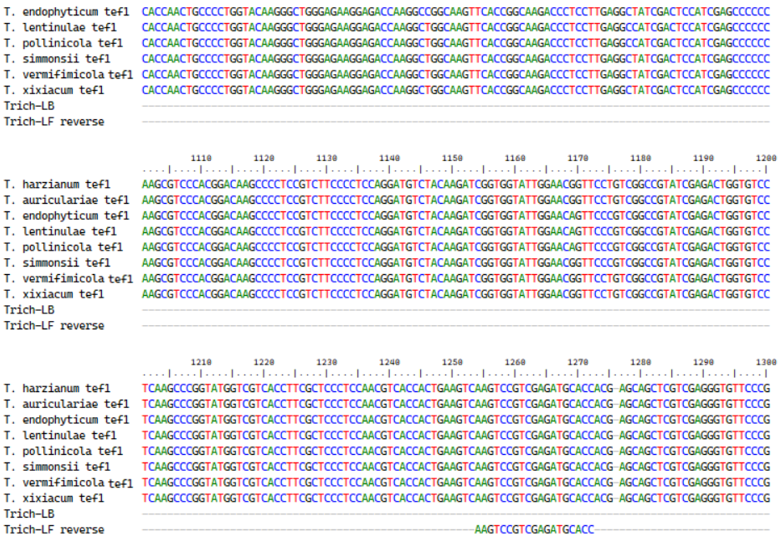
**

**
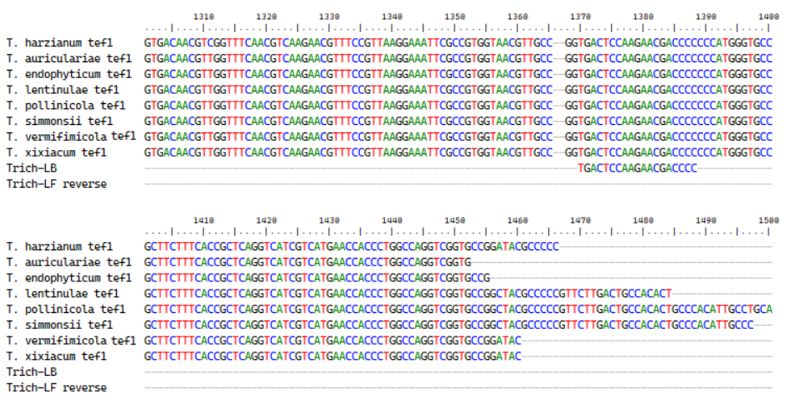
**

**Supplementary Data S2.** Sequence alignment done in BioEdit v7.2.0 software (Informer Technologies, Inc.) to confirm genus level specificity based on complementarity of LAMP primer sequences with homolog target regions of *tef1* gene sequences of different *Trichoderma* species: *T. harzianum* *-* GenBank: OL435125, *T. auriculariae* - GenBank: OR548070, *T. endophyticum* - GenBank: KX689257, *T. lentinulae* - GenBank: OP832395, *T. pollinicola* - GenBank: MF939621, *T. simmonsii* - GenBank: OP562772, *T. vermifimicola* - GenBank: MN605882, *T. xixiacum* - GenBank: MN605885.

1. Alignment of *tef1* gene sequences of *Trichoderm*a species with F3 and B3 (reverse complementary) primer sequences
2. Alignment of *tef1* gene sequences of *Trichoderm*a species with the regions of FIP (F1c+F2) primer sequence (F1c reverse complementary sequence and F2 sequences separately aligned)
3. Alignment of *tef1* gene sequences of *Trichoderm*a species with the regions of BIP (B1c+ B2) primer sequence (B1c sequence and B2 reverse complementary sequence separately aligned)
4. Alignment of *tef1* gene sequences of *Trichoderm*a species with LF (revers complementary) and LB primer sequences

**
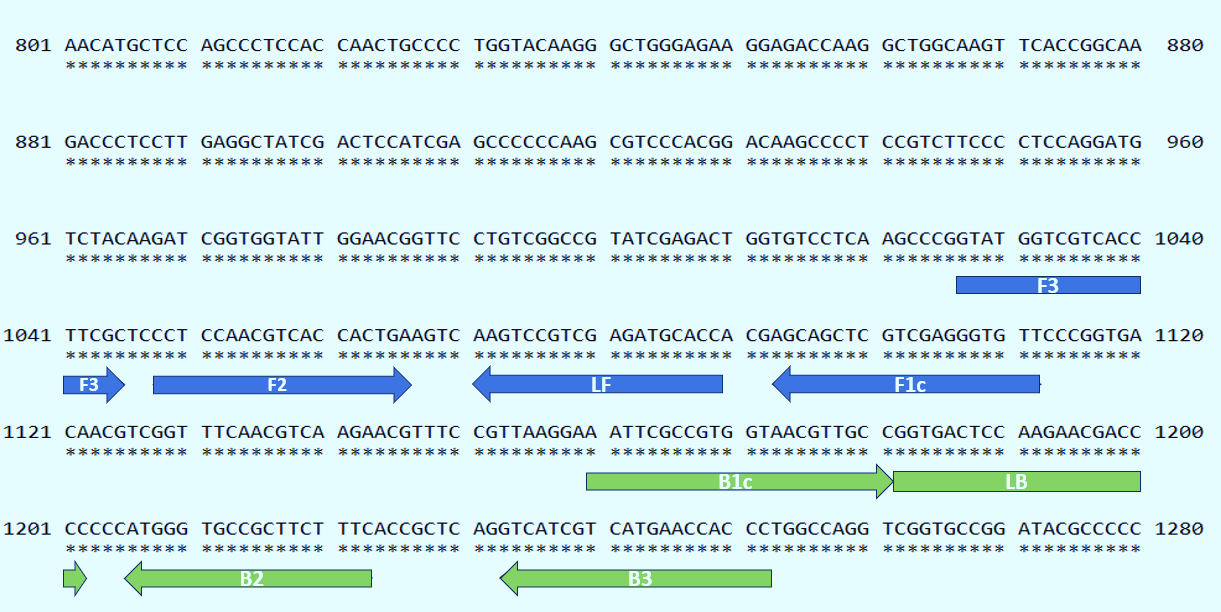
**

**Supplementary Figure S1.** The position and direction of all LAMP primers within the *- GenBank* gene (*tef1* gene of *Trichoderma harzianum*; GenBank: OL435125).

**Supplementary Figure S2**. Absorbance spectrum of synthetized AuNPs.

**Supplementary Table S1.** Calculated parameters of the synthetized AuNPs obtained using their absorption spectrum in Supplementary Figure S2 (Haiss et al., 2007).

**
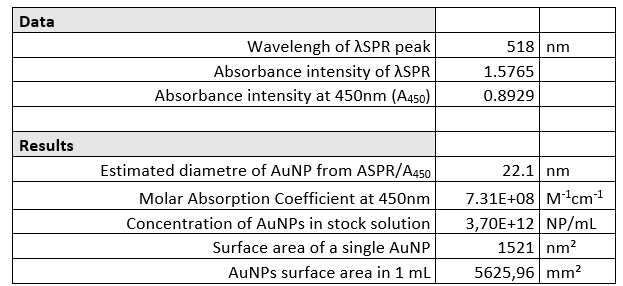
**


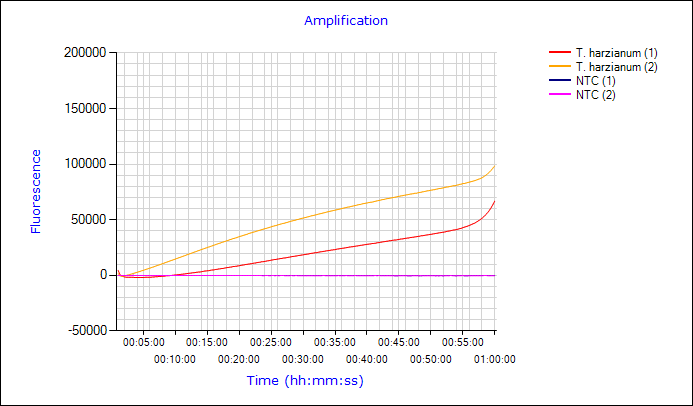


**Supplementary Figure S3.** LAMP optimization – testing the reaction at 60 ℃ during 60 minutes.


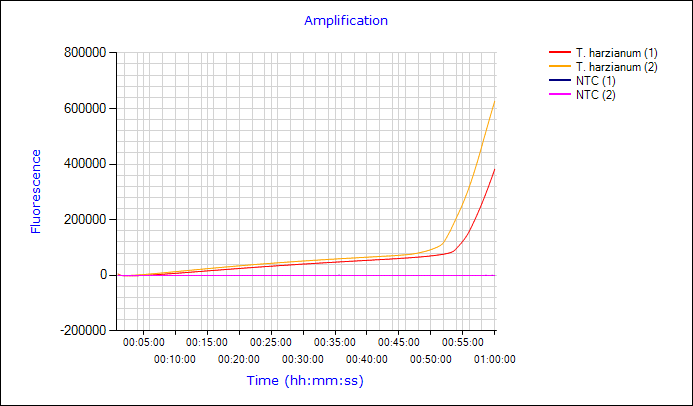


**Supplementary Figure S4.** LAMP optimization – testing the reaction at 62 ℃ during 60 minutes.


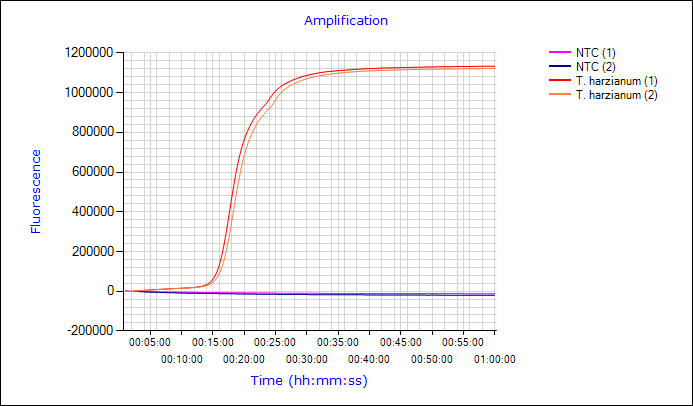


**Supplementary Figure S5.** LAMP optimization – testing the reaction at 65 ℃ during 60 minutes.


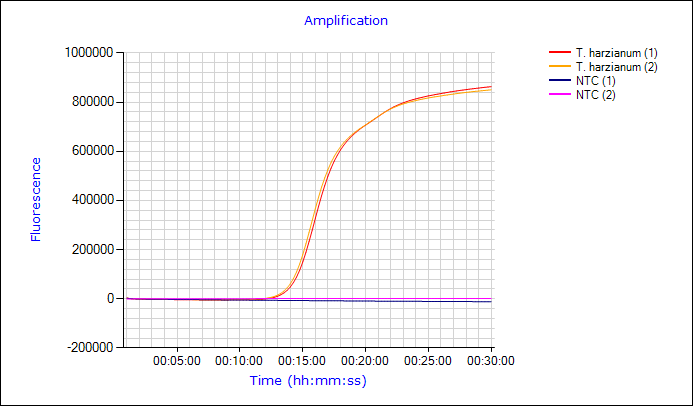


**Supplementary Figure S6.** LAMP optimization – testing the reaction during 30 minutes.


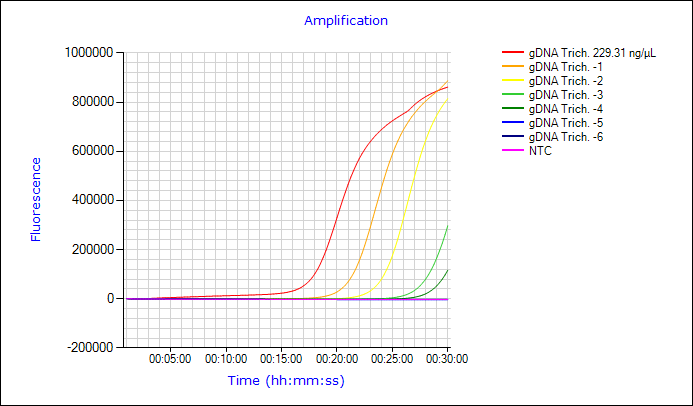


**Supplementary Figure S7.** LAMP assay sensitivity testing.

**
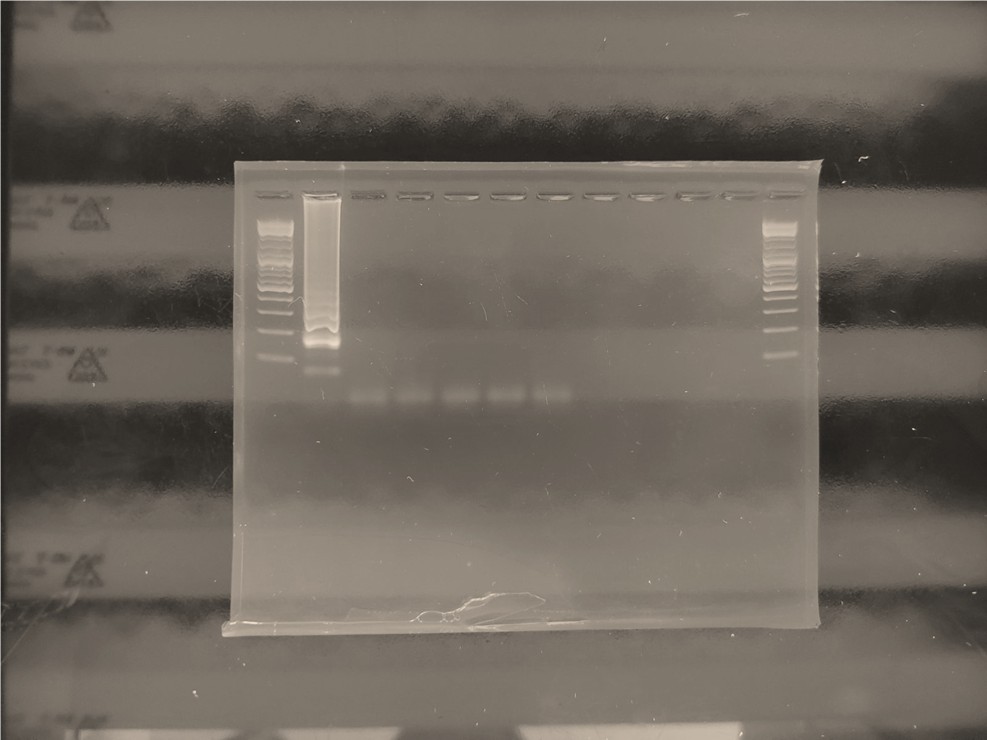
**

**Supplementary Figure S8.** An image of the full-size, uncropped 2% agarose gel used in this study. An image of the cropped gel can be found in Figure 2B within the manuscript.

**References:**

Haiss, W., Thanh, N. T. K., Aveyard, J., & Fernig, D. G. (2007). Determination of Size and Concentration of Gold Nanoparticles from UV−Vis Spectra. *Analytical Chemistry*, *79*(11), 4215–4221. https://doi.org/10.1021/ac0702084
